# Practice harmonization workshops of EBMT: an expert-based approach to generate practical and contemporary guidelines within the arena of hematopoietic cell transplantation and cellular therapy

**DOI:** 10.1038/s41409-023-01958-w

**Published:** 2023-03-27

**Authors:** Ibrahim Yakoub-Agha, Raffaella Greco, Francesco Onida, Rafael de la Cámara, Fabio Ciceri, Selim Corbacioglu, Harry Dolstra, Bertram Glass, Michelle Kenyon, Donal P. McLornan, Bénédicte Neven, Regis Peffault de Latour, Zinaida Peric, Annalisa Ruggeri, John A. Snowden, Anna Sureda, Isabel Sánchez-Ortega

**Affiliations:** 1 Chair of the Practice Harmonization and Guidelines committee of EBMT, Barcelona, Spain; 2 CHU de Lille, Univ Lille, INSERM U1286, Infinite,, 59000 Lille, France; 3 Co-Chair of the Practice Harmonization and Guidelines committee of EBMT, Barcelona, Spain; 4 Chair of the ADWP of the EBMT, Barcelona, Spain; 5 Hematology and BMT Unit, IRCCS San Raffaele Hospital, Milano, Italy; 6 Co-Chair of the Practice Harmonization and Guidelines Committee of the EBMT, Barcelona, Spain; 7 Hematology and BMT Centre, Fondazione IRCCS Ca’ Granda Ospedale Maggiore Policlinico, University of Milan, Milan, Italy; 8 Scientific Council Representative with the Registry Portfolio and Chair of the IDWP of EBMT, Barcelona, Spain; 9 Department of Haematology, Hospital de la Princesa, Madrid, Spain; 10 Chair of ALWP of EBMT, Barcelona, Spain; 11 Vita-Salute San Raffaele University, IRCCS San Raffaele Scientific Institute, Milano, Italy; 12 Scientific Council Chair with the Research and Sciences Portfolio and Chair of the PDWP of EBMT, Barcelona, Spain; 13 Department of Pediatric Hematology, Oncology and Stem Cell Transplantation, University Franz-Josef-Strauss-Allee, Regensburg, Germany; 14 EBMT Treasurer, Barcelona, Spain; 15 Department of Laboratory Medicine, Radboud University Medical Centre, Nijmegen, The Netherlands; 16 Chair of LWP of EBMT, Barcelona, Spain; 17 Helios Klinik Berlin-Buch, Berlin, Germany; 18 President Nurses Group of EBMT, Barcelona, Spain; 19 Department of Haematological Medicine, King’s College Hospital NHS Foundation Trust, London, UK; 20 Chair of the CMWP of EBMT, Barcelona, Spain; 21 Department of Haematology and Stem Cell Transplantation, University College London Hospitals NHS Trust, London, NW1 2PG UK; 22 Chair of the IEWP of EBMT, Barcelona, Spain; 23 Unité d’Immuno-hematologie et Rhumatologie Pédiatrique, Hôpital Necker-Enfants Malades, Assistance Publique Hopitaux de Paris, Université de Paris, Paris, France; 24 Scientific Council Co-chair with the Research and Sciences Portfolio and Chair of the SAAWP of EBMT, Barcelona, Spain; 25 French Reference Center for Aplastic Anemia and Paroxysmal Nocturnal Hemoglobinuria, BMT Unit, Saint-Louis Hospital and Université de Paris Cité, Paris, France; 26 Chair of the TCWP of EBMT, Barcelona, Spain; 27 School of medicine, University of Zagreb and University Hospital Centre Zagreb, Zagreb, Croatia; 28 Chair of the CTIWP of EBMT, Barcelona, Spain; 29 EBMT Secretary and Member JACIE committee, Barcelona, Spain; 30 Sheffield BMT & Cellular Therapy Programme, Department of Haematology, Sheffield Teaching Hospitals NHS Foundation Trust, Sheffield, UK; 31 EBMT President, Barcelona, Spain; 32 Institut Català D’Oncologia - Hospital Duran i Reynals, IDIBELL, Universitat de Barcelona, Barcelona, Spain; 33 Secretary of the Practice Harmonization and Guidelines committee of EBMT, Barcelona, Spain; 34 EBMT Medical Officer, Executive Office, Barcelona, Spain

**Keywords:** Health services, Health care

## Abstract

For hematopoietic cell transplantation (HCT) and cellular therapy (CT), clinical patient care is localized, and practices may differ between countries and from center to center even within the same country. Historically, international guidelines were not always adapted to the changing daily clinical practice and practical topics there were not always addressed. In the absence of well-established guidelines, centers tended to develop local procedures/policies, frequently with limited communication with other centers. To try to harmonize localized clinical practices for malignant and non-malignant hematological disorders within EBMT scope, the practice harmonization and guidelines (PH&G) committee of the EBMT will co-ordinate workshops with topic-specific experts from interested centers. Each workshop will discuss a specific issue and write guidelines/recommendations that practically addresses the topic under review. To provide clear, practical and user-friendly guidelines when international consensus is lacking, the EBMT PH&G committee plans to develop European guidelines by HCT and CT physicians for peers’ use. Here, we define how workshops will be conducted and guidelines/recommendations produced, approved and published. Ultimately, there is an aspiration for some topics, where there is sufficient evidence base to be considered for systematic reviews, which are a more robust and future-proofed basis for guidelines/recommendations than consensus opinion.

## Introduction

In the last two decades, the hematopoietic cell transplantation (HCT) and cellular therapy (CT) arenas have witnessed significant developments in both conditioning regimens and stem cell sourcing in addition to the available management options for post-treatment complications. Moreover, more novel cellular therapy approaches have rapidly emerged and new disease indications are currently expanding the application of HCT and CT in clinical practice.

This growth has resulted in a growing heterogeneity in practices across transplant and cellular therapy centers not only in Europe, but also globally. Despite the significant international efforts of national and international conferences aiming to build consensus within the field, many questions still require scientifically validated or expert consensus based and accepted practical answers. One relevant approach to effectively improve outcomes for patients undergoing HCT and CT treatments is by disseminating knowledge to transplant and non-transplant scientific communities, patient organizations and the lay public.

Therefore, the practice harmonization and guidelines (PH&G) committee, as part of the European Society for Blood and Marrow Transplantation (EBMT), has been presented to the executive committee and approved by the board of the EBMT. This committee will promote and conduct annual workshops with the aim of harmonizing HCT and CT clinical practice and standardizing procedures within EBMT, to find a common ground between the EBMT centers that wish to participate in workshops focused on areas where the literature has yet to provide clear guidance. Table 1 depicts the composition of the EBMT PH&G committee.

**Table 1:** Composition of the EBMT PH&G committee.

Administration: • Chair • Two co-chairs including the scientific council representative with the education portfolio of EBMT • secretary • Data manager/study coordinator
Permanent members: • President of EBMT • EBMT working party chairs depending on the subject

### The legacy of the SFGM-TC clinical practice harmonization workshops

The concept for the EBMT PH&G committee Workshops builds upon the legacy of the SFGM-TC (French Society for Bone Marrow Transplantation and Cellular Therapy) Harmonization Workshops that are held annually to address clinically orientated practical questions in the French transplant center context. Many of the heads of the EBMT registered French transplant centers felt that they lacked clear answers on all subjects and agreed to organize these workshops that aimed to deliver clear answers [1]. The first SFGM-TC workshops were held in Lille in September 2010 and have been held annually to build upon the discussions from previous years. Each year 10–15 workshops were held [2–32].

### The EBMT clinical practice harmonization workshops

#### Workshop setup

The PH&G EBMT workshops will be based on the successful SFGM-TC model. Each workshop is composed of one or two group leaders and up to 6–14 participants. In the four months leading up to the two-day workshop, significant preparatory work is carried out. This work takes the form of a literature review and responses concerning clinical practice collected from national or international questionnaires distributed to participating centers [33, 34]. Each workshop addresses one or more issues about a given set of practices by providing scientifically validated answers that are supported, when appropriate, by the literature. If necessary, the input of external experts is strongly recommended, especially from laboratory biologists, non-transplant disease-specialists or other allied professionals such as psychologists. An example might be inviting an expert in Therapeutic Education and one or two psychologists to make contributions to a workshop based on patient mental health and chronic morbidity following transplant or CAR-T therapy.

The face-to-face component of the workshops will ideally take place in autumn over a two-day period. During the afternoon of the second day, preliminary results will be presented at a plenary meeting of all workshop participants. At that point, all workshop groups will have completed an initial draft of their article. These manuscripts will be then sent to an expert and comprehensive review panel who will comment, revise and give their opinion before final validation by the workshop leaders. The articles will be then submitted for publication.

#### Workshop yearly publication timeline

**Fig. 1:**
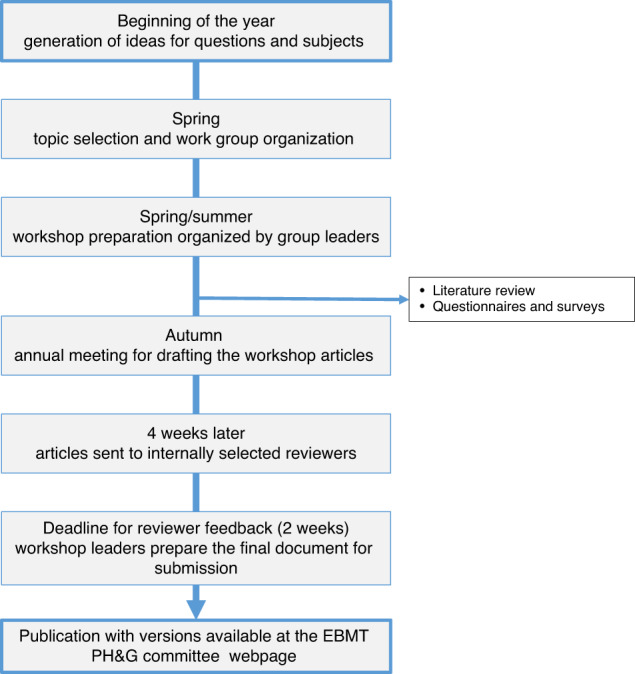
Timeline of the EBMT practice harmoninzation and guidelines workshops.

Figure 1 shows a timeline of the publication process. This process will begin in the beginning of the year with the collection of topics to be addressed and will end with the publication of the guidelines within 3–6 months after the two-day meeting where the initial manuscripts have been drafted.

#### Generating ideas

Starting at the beginning of each year, the PH&G committee will send an e-mail to all EBMT working party chairs asking for ideas on the topics that members wish to address in future workshops. Working party chairs, together with the working party active members of the involved subcommittee (depending on the specific proposed topic), suggest potential participants as well as potential external experts for each workshop. Having received ideas, potential research questions are classified into categories and prepared for the next step.

#### Refining topics and organizing workshop groups

The EBMT PH&G committee selects 10–12 questions/topics by trying to maintain a diversity covering all categories of the workshops i.e., five clinical questions addressing adult patients, one question for pediatric patients, one question for treatment co-ordination, questions for different disease indications when appropriate, one question for data management, one question for HLA biology or other relevant laboratory issues and one question for cell therapy practices. Exceptionally, it is possible to add a question if the current situation requires it (for example, a new indication for CAR T-cell therapy). It is also possible to rework an issue from previous years to update guidelines in response to recent progress in the field.

After approval by the PH&G committee, the secretary of the committee will send an e-mail to all possible participants so that they can select a maximum of three workshops in order of preference. It is important to note that participants who are not active members of the EBMT can still participate in the workshops if their input is relevant to the work of a certain group.

Given the responses, the committee members form the working groups. Each group will consist of one or two leaders. The number of participants varies depending on the subject and the selection that members have made. As mentioned before, external experts may be invited as appropriate. Table 2 summarizes the composition of the working group.

**Table 2:** Composition of a workshop.

	Number	Role
Workshop leaders	1–2	Leading the workshop, organizing the pre-meeting work. Drafting and validation and submission of the final manuscript
Participants	6–18	Preparation of the work and recommendations
EBMT PH&G committee members	All	Selection the topic, validation of the workshop composition, providing help in the whole process and validation of the final manuscript
External experts	0–2	Participation to the workshop and providing expertise
Internal reviewers	0–2	Review of the final draft

#### Aspiration for systematic reviews

Ultimately, there is an aspiration for some topics where there is sufficient evidence base to be considered for systematic reviews, which are accepted as a more robust and future-proofed basis for guidelines and recommendation compared with consensus opinion. The EBMT has current guidelines [35, 36]. However, systematic reviews require additional expertise and resource which will require individual consideration. Ideas for systematic reviews will be welcomed by the PH&G Committee and appropriate representation will be made to EBMT for resourcing individual projects.

#### Selection of group leaders and external experts

For each group, one or two leaders will be appointed. As needed, one or two potential external experts may be invited to join the group according to the topic.

Experts and leaders are selected based on:

- relevant professional experience in areas related to the topic
- previous research and scientific publications in the relevant topic
- availability to meet face-to-face for the Autumn two-day workshop and virtually during the process.
- EBMT Equality, Diversity and Inclusion (EDI) position statement and policies [37]. EDI is central to the mission, vision and values of EBMT and the selection of group leaders and external experts will consider principles of EDI, particularly in avoiding conscious and unconscious bias. In addition, there can be exceptional consideration of the personal circumstances of experts and their ability to meet face to face versus virtually throughout the process.

#### Pre-workshop preparation

Workshop leaders organize the necessary research to be done before the workshop by organizing conference calls or in-person meetings with their group participants. This work takes the form of an exhaustive literature review carried out by the workshop participants in addition to data collection via questionnaires and surveys. Bespoke questionnaires and surveys can be circulated amongst centers to evaluate practices and tailor final recommendations to suit center-specific needs. A large part of the research is to be completed before the autumn meeting.

#### The clinical practice harmonization workshops

The two-day workshop, which takes place in Autumn, is organized into four half-days, the first three of which are devoted to drafting the basic document that gets sent to the first round of reviewers. Once the article is written, a PowerPoint or equivalent presentation must also be prepared for the plenary session at the end of the second day. Each workshop group leader and one or two group participants present the results from their workshop.

The document and the presentation should be organized as follows:

- Questions asked/issues to be addressed
- Current state of the art
- Methodology
- Workshop recommendations
- Unanswered questions and further research to do in the field
- References (needed for the document only)

#### Revision process

The workshop coordinator will collect the articles at the end of the second day. After retrieving the basic documents, they will be sent to internally selected reviewers for expert feedback.

#### Preparing the final article

The workshop leaders will be responsible for the preparation of the final manuscripts according to the format required by the chosen journal. The title should be followed by “recommendations from the EBMT practice harmonisation and guidelines committee”. The EBMT PH&G committee will be responsible for finding PubMed referenced journals in order to ensure a wide dissemination of the recommendations of the EBMT PH&G committee and to increase the visibility of the organization.

#### Order of co-authors

The workshop leaders take the place of first and last author. In the absence of a specific group leader, participant names will be placed alphabetically followed by the names of the PH&G committee’s members, the selected reviewers and external experts in alphabetical order. The EBMT PH&G committee chair, on the other hand, will automatically be the second-to-last author on all articles unless he/she is him/herself the workshop leader.

As clinical practice guidelines published by a scientific association and not scientific articles, workshop leaders must be first and last author to insure the authority of each set of recommendations. As a result, our younger peers will not be allowed to be first author for the sole purpose of having a “first author” publication. If a workshop leader is tempted to make this gesture, it will be acceptable to let the younger peer be second author.

#### Print and online publication

If the submitted articles are published without a strict peer review process of external reviewers, the committee members will check all the articles before submission as part of an internal validation process. It is imperative that finalized manuscripts are formatted according to the guide for authors of the journal that will be publishing the special issue. The final version of the article will be sent to the secretariat of the EBMT PH&G Committee for validation by the committee members. If possible, it would be preferred to go for a grouped submission with the rest of the articles. The submission process will be the responsibility of the workshop coordinator. If the coordinator detects any issue with the research conducted, he/she should discuss the issues with the workshop leaders before making necessary changes.

In addition to having a PubMed referenced publication, the article manuscripts will be immediately posted online for immediate clinical access and use via the EBMT PH&G committee webpage. On occasion, brochures of a specific set of guidelines may be made for easy distribution and use in EBMT centers.

## Data Availability

Data sharing not applicable to this article as no datasets were generated or analysed during the current study.
